# THE EXPERIENCE OF HAPPINESS IN LATE-LIFE REPARTNERING: SURPRISE AND DISAPPOINTMENT

**DOI:** 10.1093/geroni/igz038.2138

**Published:** 2019-11-08

**Authors:** Chaya Koren

**Affiliations:** University of Haifa, Haifa, Israel, Israel

## Abstract

Late life repartnering among those aged 65 and older is a phenomenon developing along with the increase in life expectancy. Although research indicates that older people are happier than adults at other life phases, the common lay person perception among the young as well as the old is that old age is associated with less happiness. Late life repartnering in Israel, culturally located between tradition and modernity, is not officially recognized as an option in old age. Exploring the experience of happiness within a social context that perceives late-life repartnering as the exception, using a naturalistic paradigm, has the potential for understanding lay persons perceptions of happiness. The aim of this qualitative research is to explore the experience of happiness in late-life repartnering relationships from a dyadic perspective of each and both partners. 20 couples (40 participants) functionally independent, aged 66-92 who entered their late-life repartnership at old age (men aged 65+; women aged 60+) after widowhood or divorce from a lifelong marriage raising a family, were interviewed separately. Interviews were recorded and transcribed verbatim. Data was analyzed using a dyadic interview analysis method. Findings indicate that happiness in late-life repartnering relationships include experiences of surprise and disappointment in three sub-themes: a. “A gift from heaven”: Surprised of being happy; b. Disappointment not being happy; c. No surprise – No disappointment. Findings are discussed based on disappointment theory, and empirical literature on expectations and happiness. Implications are addressed.

